# Depression, Anxiety, and Stress among Hangover-Sensitive and Hangover-Resistant Drinkers

**DOI:** 10.3390/jcm12082766

**Published:** 2023-04-07

**Authors:** Andy J. Kim, Agnese Merlo, Marlou Mackus, Gillian Bruce, Sean J. Johnson, Chris Alford, Simon B. Sherry, Sherry H. Stewart, Joris C. Verster

**Affiliations:** 1 Department of Psychology and Neuroscience, Dalhousie University, 1355 Oxford St., Halifax, NS B3H 4R2, Canada; 2 Division of Pharmacology, Utrecht Institute for Pharmaceutical Sciences, Utrecht University, 3584CG Utrecht, The Netherlands; 3 Division of Psychology, School of Education and Social Sciences, University of the West of Scotland, Paisley PA1 2BE, UK; 4 Centre for Trials Research, Cardiff University, Cardiff CF14 4YS, UK; 5 Centre for Human Psychopharmacology, Swinburne University, Melbourne, VIC 3122, Australia; 6 Department of Psychiatry, Dalhousie University, 5909 Veterans’ Memorial Lane, Halifax, NS B3H 2E2, Canada

**Keywords:** alcohol, hangover, resistance, anxiety, stress, depression

## Abstract

This study investigated potential differences in baseline (i.e., non-hangover-related) levels of depression, anxiety, and stress between individuals who are sensitive to and those resistant to hangovers after consuming alcohol. Participants included 5111 university students from the Netherlands and the U.K., including 3205 hangover-sensitive and 1906 hangover-resistant drinkers. All participants completed surveys on their demographics, alcohol consumption, and hangover susceptibility (whether they experienced a hangover in the past 12 months), as well as their baseline levels of depression, anxiety, and stress on the DASS-21 scale. The results showed that hangover-sensitive drinkers had significantly higher levels of anxiety and stress, but not depression, compared to hangover-resistant drinkers. However, the observed differences between the two groups were small, with a magnitude of less than 1 out of 42 points on the DASS-21 anxiety and stress subscales, and are thus unlikely to be clinically meaningful.

## 1. Introduction

Alcohol hangover refers to the combination of negative mental and physical symptoms which can be experienced after a single episode of alcohol consumption, starting when blood alcohol concentration (BAC) approaches zero [1]. The majority of individuals (approximately 80%) are sensitive to hangovers after drinking [2,3,4]. However, a significant minority (approximately 20%) do not experience hangovers and are considered to be resistant to them [5]. Understanding the factors that can distinguish hangover-sensitive individuals from those who are resistant can potentially provide insight into the mechanisms underlying alcohol hangovers [6].

Individual differences in susceptibility to alcohol hangovers are not fully understood. Researchers have shown that the presence and severity of hangovers are related to an immune response to alcohol, in which the inflammatory effects of alcohol persist and manifest as hangover symptoms the following day [7,8].

Another potential biological explanation for individual differences in hangover susceptibility pertains to differences in the rate at which ethanol is metabolized [9], suggesting that individuals with a faster ethanol elimination rate may experience less severe hangovers [10]. Research has shown that hangovers can be experienced after consuming any amount of alcohol, depending on the individual drinker [11]. Thus, while biological factors play a role in the pathology of the alcohol hangover, further research is needed to explain why some individuals experience hangovers after drinking and others do not (i.e., individual differences in hangover susceptibility). In particular, psychosocial factors, such as emotional wellbeing, deserve further study in terms of their potential role in hangover sensitivity.

There is a known link between emotional wellbeing and drinking behaviors. For example, research has shown that individuals with higher levels of depression, as measured by the DASS-21 scale, tend to drink more heavily over time, while those with higher levels of anxiety, as measure by the same scale, tend to drink less heavily [12]. Additionally, individuals who use alcohol to cope with depression and anxiety may be more likely to experience negative consequences related to alcohol use, such as blacking out [13]. Given these connections between emotional wellbeing and alcohol use, it is possible that hangover pathology may also be related to emotional wellbeing.

The current literature on the links of emotional wellbeing with hangover susceptibility is limited. In one study of young adults, a validated measure of overall mood in the non-hangover state, which included items pertaining to depression, anxiety, and stress, was not a significant predictor of hangover severity [14]. However, this study did not differentiate between hangover-sensitive and hangover-resistant drinkers. Although not designed to examine hangover susceptibility, a recent study did not find significant differences in non-hangover-related anxiety or depression, as measured by the DASS-21, between university student drinkers who reported experiencing a hangover at a 2-week follow-up versus university student drinkers who did not report a past 2-week hangover [15]. However, this study allocated drinkers to these two hangover groups based on experiences over a short 2-week period. This may not have accurately captured hangover susceptibility as a sufficient drinking episode may not have occurred during this period. Additionally, the study was limited by a small sample size (e.g., *n* = 97 no-hangover- and *n* = 39 hangover-reporting participants), suggesting a need for replication and extension with a longer period of reference for the hangover experience and a larger sample size.

Against this background, the present study investigated a large sample of university student drinkers. It compared baseline (i.e., non-hangover-related) levels of depression, anxiety, and stress, as measured by the DASS-21, between participants who reported experiencing a hangover in the past 12 months (hangover-sensitive) and those who did not report experiencing a hangover in the same period (hangover-resistant). Given the limited nature of existing literature on this topic, no specific hypothesis was formulated regarding which hangover group would report higher levels of depression, anxiety, or stress.

## 2. Methods

For the current analysis, data from three studies were combined [16,17,18]. These studies were conducted online using SurveyMonkey, and university students were invited to participate. The age range for participants in the Dutch studies (Study 1 [17]; Study 3 [16]) was 18 to 30 years old, while in the U.K. study (Study 2 [18]), the age range was 18 to 35 years old. No other inclusion or exclusion criteria were applied. Ethics board approval was obtained for each study, and all participants provided informed consent. A detailed description of the methodology of these studies can be found in the respective published articles [16,17,18]. Data from participants who consumed alcohol and who completed questions on alcohol consumption, hangover susceptibility, and depression, anxiety, and stress were considered for the current analysis.

### 2.1. Allocation to the Hangover-Sensitive or Hangover-Resistant Group

Participants were classified as hangover-resistant if they reported not having experienced an alcohol hangover in the past 12 months. This was verified using the Brief Young Adult Alcohol Questionnaire [19,20], which contains a single item asking whether participants have experienced a hangover in the past 12 months, with responses of “yes” (indicating hangover sensitivity) or “no” (indicating hangover resistance).

### 2.2. Past 30 Days’ Alcohol Consumption

For the heaviest drinking occasion within the past 30 days, data on the number of alcoholic drinks consumed (“In the past 30 days, what is the greatest number of alcoholic drinks you had on one occasion?”) and the duration of drinking (“On that occasion, how many hours did you consume alcohol?”) were collected [17]. A graphic was provided to illustrate the sizes of alcoholic drinks and how to convert them into standard units of alcohol (10 g). The estimated blood alcohol concentration (BAC) for this drinking occasion was calculated using a modified Widmark equation, taking into account the following factors: sex, bodyweight, amount of alcohol consumed, and duration of the drinking session [21].

### 2.3. Depression, Anxiety, and Stress

The 21-item version of the Depression Anxiety Stress Scales (DASS-21), consisting of three subscales for depression, anxiety, and stress (each with 7 items), was utilized for this study [22,23]. The items were scored on a 4-point Likert scale (from 0 = not at all to 3 = very much or most of the time). The scores for each subscale were totalled (out of 21) and then multiplied by 2 to allow for comparison with the DASS-42 and other published DASS-21 data [24]. A maximum score of 42 on each subscale signifies an extremely severe level of depression, anxiety, or stress. The DASS-21 has been found to be a reliable and valid measure, with support for its three-factor structure [25,26]. In Studies 1 and 2 [17,18], the DASS-21 was a momentary assessment (no time period specified), while in Study 3 [16], the DASS-21 referred to the past 4 weeks.

### 2.4. Statistical Analysis

Statistical analyses were conducted with R (Version 4.2.1). Basic descriptive statistics were calculated for each variable, including the mean and standard deviation (*SD*). Data were not normally distributed, so the Kruskal–Wallis and Mann–Whitney U tests were used to compare the outcomes of the three studies. Chi-squared tests were used for dichotomous variables, such as sex and hangover status. A Bonferroni adjustment (*p* < 0.017) was applied to account for multiple comparisons. The datasets from all three studies were combined for analysis. The outcomes of the hangover-sensitive and hangover-resistant group were compared again using the Mann–Whitney U test or the chi-squared test for dichotomous data. In case demographic variable data were missing for a participant (e.g., age, body weight), the participant was not included in the corresponding analysis. The overall number of missing observations was minimal (0.15%).

## 3. Results

The combined dataset included 5111 participants. Their demographics and study outcomes appear in Table 1. Most participants were hangover-sensitive females. On their heaviest drinking occasion in the past 30 days, participants reported consuming an average of 8.2 (*SD* = 6.1) alcoholic drinks over 5.2 (*SD* = 3.0) hours. There were statistically significant differences across the three studies in terms of age, the proportion of females and hangover resistance, body weight, number of alcoholic drinks consumed, drinking duration, estimated BAC, and all subscales of the DASS-21.

**Figure 1:**
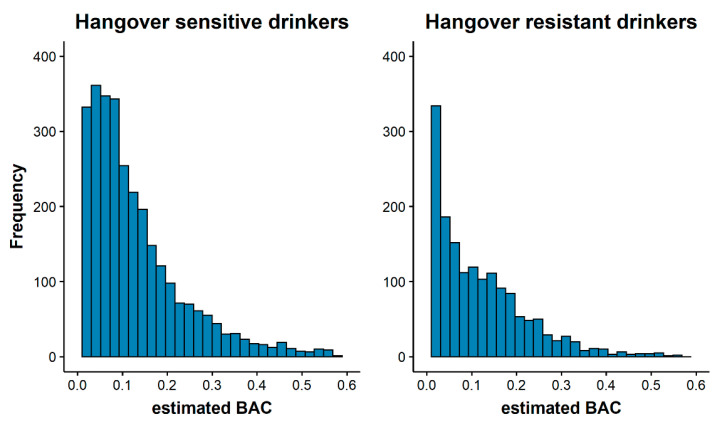
Distribution of estimated BAC for hangover-sensitive and -resistant drinkers. Abbreviation: estimated BAC = estimated blood alcohol concentration.

Figure 1 illustrates the distribution of the estimated BAC for both groups. Both hangover-sensitive and -resistant drinkers showed a similar trend, with most participants having an estimated BAC below 0.10% for their heaviest drinking occasion in the past month and fewer participants in each subsequent higher estimated BAC group. 

Table 2 presents a summary of the data for hangover-sensitive and -resistant drinkers in the combined dataset. The scores for depression, anxiety, and stress were within the expected range based on the means derived from normative samples [27]. Hangover-sensitive drinkers had significantly higher anxiety and stress scores compared to hangover-resistant drinkers with mean differences of 0.4 and 0.6, respectively. However, these mean differences were inconsequential (i.e., too small to be clinically meaningful), with effect sizes of r = 0.05 for anxiety and r = 0.03 for stress. Furthermore, there was no significant difference between the two groups in terms of the depression scores. Hangover-sensitive drinkers also reported consuming a significantly greater number of alcoholic drinks, drinking for a longer duration, and having a higher estimated BAC on their heaviest drinking occasion in the past 30 days compared to hangover-resistant drinkers. These were all small effects (See Table 2). Additionally, there was a higher proportion of females in the hangover-resistant group (74.13%) compared to the hangover-sensitive group (68.83%).

As a sensitivity test, the DASS-21 subscale scores for hangover-sensitive and -resistant drinkers were reanalysed using negative binomial regression models, with depression, anxiety, and stress as separate outcomes. The models included hangover status (sensitive or resistant), age, sex (male or female), body weight, and estimated BAC for the heaviest drinking occasion in the past 30 days as predictors, as well as the study (Study 1, 2, or 3). The number of alcoholic drinks and drinking duration were not included to avoid potential multicollinearity given the inclusion of the estimated BAC. The results again showed that hangover-sensitive drinkers had significantly higher levels of anxiety and stress (but not depression) than hangover-resistant drinkers, even after controlling these other potential confounders. The estimated marginal mean differences were 0.9 and 0.6, for anxiety and stress, respectively. The predictors in the model together explained approximately 9% of the variability in anxiety (Nagelkerke’s pseudo *R*^2^ = 0.09) and about 6% of the variability in stress (Nagelkerke’s pseudo *R*^2^ = 0.06), but only about 3% of the variability in depression (Nagelkerke’s pseudo *R*^2^ = 0.03).

## 4. Discussion

This study investigated the relationship between emotional wellbeing and hangover susceptibility in a large sample of university students. The analysis showed that hangover-sensitive drinkers had statistically significantly higher levels of anxiety and stress compared to hangover-resistant drinkers. The DASS-21 subscale scores between the two hangover groups showed mean differences of 0.4 and 0.6 for anxiety and stress, respectively, in the main analysis and 0.9 and 0.6 in the sensitivity test after accounting for covariates. These mean differences for anxiety and stress were however lower than the minimum change in scores found for individuals within the normative population to indicate improvement after receiving intervention (e.g., 3.9 for anxiety and 4.9 for stress) [24]. Additionally, the mean DASS-21 subscale scores for both hangover-sensitive and hangover-resistant drinkers were lower than the cut-off scores for the severity ratings [23], indicating mild levels of depression (10), anxiety (8), and stress (15). Finally, in the main analysis, the effect sizes for the mean differences between the two hangover groups for anxiety and stress were inconsequential (r = 0.05 and 0.03, respectively). This suggests that, while the differences between hangover-sensitive and -resistant drinkers were statistically significant due to the large sample size, they were not likely meaningful in terms of clinical significance.

Although emotional wellbeing may influence certain drinking behaviors, such as heavy drinking and drinking to cope [12,13], this study suggested it does not necessarily mean that individuals reporting greater depression, anxiety, or stress are more likely to experience hangovers. This may be because hangovers can occur after any level of alcohol consumption and are not necessarily linked to heavy drinking [11]. While the present data showed that hangover-sensitive individuals may consume slightly more alcohol than hangover-resistant individuals, the distribution of the estimated BAC levels were comparable between the two hangover groups (see Figure 1) and were controlled for in the sensitivity test, further indicating no meaningful differences in anxiety and stress among those who experience hangovers after drinking and those who do not.

These results further the understanding of studies showing nonsignificant associations of hangover susceptibility with mental resilience and psychological wellbeing [28], by suggesting hangover susceptibility is also not related in a clinically meaningful way to emotional wellbeing in the non-hangover state. Nonetheless, research has found that a significant minority of individuals who are sensitive to hangovers report experiencing symptoms of depression (29.9%) and anxiety (18.3%) during the hangover state [29] (a phenomenon popularly referenced in (social) media as “hangxiety” [30]); individuals reporting greater levels of depression and anxiety during the non-hangover state have been shown to be more vulnerable to experiencing depression and anxiety during a hangover [15], suggesting a possible role for emotional wellbeing in at least some aspects of the hangover experience. Moreover, this study focused on hangover susceptibility (a dichotomous variable), rather than hangover severity (a continuous measure); it is still possible that emotional wellbeing variables may be clinically meaningful predictors of more intense/aversive hangover experiences. Overall, additional research is needed to further clarify the relationship between emotional wellbeing and hangovers, as well as the role of other psychosocial factors in determining hangover susceptibility, including variables such as personality and lifestyle.

The strengths of this study include the use of validated measures of depression, anxiety, and stress, as well as an acceptable method for identifying hangover susceptibility. Additionally, the large sample size and the inclusion of samples from different countries increased the power of the study and allow for generalizability to the young adult drinking population. However, this study has limitations that should be acknowledged. One issue is that self-report surveys may be prone to recall bias, as participants may not accurately remember certain events or details. Another limitation is that the sample in this study consisted of young adults recruited from universities, which means that the results may not be generalizable to other populations, such as community adults over the age of 35 or individuals with clinical diagnoses of depression, anxiety, and/or stress disorders. Therefore, it would be useful to replicate this study with a more diverse community sample, and in clinical samples, to improve the generalizability of the findings.

## 5. Conclusions

Investigating the role of psychosocial variables such as emotional wellbeing in hangover susceptibility is important as it can help clarify the underlying mechanisms of alcohol hangovers. The current study, which included a large sample of university students from different countries, found there were no clinically meaningful differences between hangover-sensitive and hangover-resistant drinkers in their depression, anxiety, or stress that are experienced in non-hangover contexts. Further research is needed to fully understand the complex processes that underlie hangover susceptibility.

## Figures and Tables

**Table 1:** Demographics and study outcomes.

Variables Assessed	Overall	Study 1 [17]	Study 2 [18]	Study 3 [16]
*N*	5111	3056	604	1451
Sex (m/f)	1492/3619	1066/1990	184/420	242/1209 *^,†^
Age (years)	21.9 (2.7)	22.1 (2.5)	22.2 (4.2) *	21.4 (2.1) *
Bodyweight (kg)	68.7 (12.3)	69.7 (12.2)	68.0 (14.1) *	67.0 (11.4) *
Hangover resistant (%)	37.3%	33.5%	24.5% *	50.6% *^,†^
Past 30 days’ heaviest drinking occasion				
Number of alcoholic drinks	8.2 (6.1)	8.4 (6.4)	7.4 (5.8) *	8.3 (5.4) ^†^
Drinking duration (h)	5.2 (3.0)	5.3 (3.1)	5.0 (3.1) *	5.2 (2.5) ^†^
Estimated BAC (%)	0.12 (0.1)	0.11 (0.1)	0.10 (0.1)	0.13 (0.1) *^,†^
DASS-21				
Depression	5.7 (7.1)	5.2 (6.6)	8.6 (9.5) *	5.5 (6.9) ^†^
Anxiety	5.5 (6.5)	4.3 (5.3)	7.2 (7.8) *	7.4 (7.5) *
Stress	8.9 (7.9)	7.8 (7.3)	12.7 (9.4) *	9.7 (7.7) *^,†^

The mean and standard deviation (*SD*) are shown. Significant differences (*p* < 0.017, after Bonferroni’s correction for multiple comparisons) are indicated by * (significant difference from Study 1) or ^†^ (significant difference from Study 2). Abbreviations: BAC = blood alcohol concentration; DASS-21 = Depression, Anxiety, and Stress Scales, 21-item version.

**Table 2:** Demographics and study outcomes of hangover-sensitive and -resistant drinkers.

Variables Assessed	Hangover-Sensitive	Hangover-Resistant	Effect Size (r)
*N*	3205	1906	
Sex (m/f)	999/2206	493/1413 *	0.06
Age (years)	21.9 (2.7)	21.8 (2.6)	0.02
Bodyweight (kg)	68.7 (12.1)	68.7 (12.6)	0.00
Past 30 days’ heaviest drinking occasion			
Number of alcoholic drinks	8.8 (6.2)	7.2 (5.8) *	0.15
Drinking duration (h)	5.5 (3.0)	4.8 (2.8) *	0.13
Estimated BAC (%)	0.12 (0.1)	0.10 (0.1) *	0.11
DASS-21			
Depression	5.8 (7.2)	5.5 (7.0)	0.03
Anxiety	5.7 (6.4)	5.3 (6.5) *	0.05
Stress	9.1 (8.0)	8.5 (7.6) *	0.03

Notes: The mean and standard deviation (*SD*) and the Wilcoxon effect sizes (r) are shown. Significant differences (*p* < 0.05) are indicated by *. Abbreviations: BAC = blood alcohol concentration, DASS-21 = Depression, Anxiety, and Stress Scales, 21-item version.

## Data Availability

The data are available upon reasonable request from the corresponding author.
